# Effect of Dietary Calcium Nitrate Addition on Methane Emission, Nitrogen Excretion, and Ruminal Fermentation Parameters and Microbiota in Liuyang Black Goats

**DOI:** 10.3390/ani16081150

**Published:** 2026-04-10

**Authors:** Mingming Li, Ting Liu, Chen Zheng, Xuan Nan, Jun Wang, Baicong Chen, Hanfang Zeng

**Affiliations:** 1 College of Animal Science and Technology, Gansu Agricultural University, Lanzhou 730070, China; 15293945390@163.com (M.L.); liuting@gsau.edu.cn (T.L.); zhengc@gsau.edu.cn (C.Z.); 2 Lanzhou Animal Disease Prevention and Control Center, Lanzhou 730046, China; nanxuan6150@163.com; 3 Animal Husbandry Station of Beijing, Beijing 100107, China; wj552510_78@163.com (J.W.); allpass0907@sina.com (B.C.)

**Keywords:** calcium nitrate, greenhouse gas, nutrient digestion, 16S rRNA, rumen fermentation

## Abstract

This research explored how dietary supplementation of calcium nitrate influences methane emissions, nitrogen excretion, ruminal fermentation parameters, and microbiota in Liuyang black goats. A total of twelve male goats from this breed were divided into two groups: one serving as a control group (CON), while the other received a treatment of 3% calcium nitrate (CAL). The research was conducted over a period of 40 days and comprised two separate trial phases. A 10-day adaptation period and a 5-day sampling period (days 11–15) for each stage. Results showed that incorporating calcium nitrate significantly reduced the emissions of methane (CH_4_) (*p* < 0.05) and carbon dioxide (CO_2_) (*p* < 0.05). Moreover, the use of calcium nitrate modified the trends in ruminal fermentation, resulting in an increase in pH (*p* < 0.05). Moreover, the ratio of acetate to propionate (A:P) was notably reduced in the CAL group (*p* < 0.05), indicating a shift toward enhanced production of propionate. At the microbial level, an increased presence of *Bacteroidota* and Prevotella was observed in the CAL group (*p* < 0.05). In contrast, the CON group exhibited elevated levels of *Firmicutes* and *Methanobrevibacter* (*p* < 0.05). This finding suggests that calcium nitrate plays a significant role in reducing methane emissions and also affects the fermentation processes in the rumen along with the microbiota of Liuyang black goats. Further research is needed to examine the long-term implications of calcium nitrate supplementation on the health and productivity of these goats.

## Simple Summary

This study explored the effects of incorporating calcium nitrate into the diets of Liuyang black goats on methane emissions and their digestive processes. Results revealed that the addition of calcium nitrate significantly reduced both methane and carbon dioxide emissions from these goats. Furthermore, three hours after feeding, an increase in rumen pH was observed, accompanied by a decrease in ammonia concentration and a reduction in the acetate-to-propionate ratio. Additionally, calcium nitrate facilitated a decline in the population of specific microorganisms within the rumen. In summary, research indicates that calcium nitrate is highly effective in reducing methane emissions in goats and optimizing their gut microbial environment. These findings provide valuable theoretical perspectives on the role of calcium nitrate in the feeding regimens of Liuyang black goats.

## 1. Introduction

Global warming stands as one of the most urgent challenges facing the world in the 21st century, primarily driven by greenhouse gases (GHGs) [1]. Notably, methane (CH_4_) is of significant relevance, given that its ability to contribute to global warming is 28 times more potent than that of carbon dioxide (CO_2_) when assessed over a period of one hundred years [2]. Ruminants, including cattle, sheep and goats, are significant sources of CH_4_ emissions due to their unique digestive process involving enteric fermentation in the rumen [3]. Enteric CH_4_ emissions, which primarily result from enteric methanogenesis, a microbial process that takes place in the digestive tract, are receiving increasing attention. This is due to their significantly higher global warming potential compared to CO_2_, as well as their significant contribution to total agricultural emissions, accounting for approximately 39% [4]. Rumen fermentation in ruminants creates optimal growth conditions and a nutrient-rich environment for microorganisms. It also enables efficient utilization of fibrous feedstuffs that are typically difficult to digest [5]. While rumen fermentation promotes fiber degradation, 2% to 15% of ingested dietary energy is concurrently lost as CH_4_. Therefore, practical feeding strategies are needed to reduce CH_4_ emissions from ruminants. Earlier research indicates that nitrate demonstrates a potential to mitigate CH_4_ levels in goats [6]. In the rumen, nitrates are reduced to nitrites and ammonia, which compete with rumen methanogenesis for reducing equivalents and reduce CH_4_ emissions [7]. Although nitrate supplementation holds potential advantages, its use in ruminant diets has been constrained by worries regarding toxicity and the significant inconsistencies in CH_4_ reduction reported in various studies. Nitrate reduction in the rumen requires an adaptation period to increase the population of nitrate-nitrite reducing bacteria, thereby preventing poisoning from nitrite accumulation [8]. Reasonable and appropriate supplementation does not adversely affect the production health of goats [9,10]. Calcium nitrate, a soluble form of nitrate, has emerged as a potential alternative that can be rapidly absorbed in the rumen and utilized by rumen microbes to produce less CH_4_-intensive fermentation products, such as propionate, thereby mitigating the problems associated with other forms of nitrate while still providing the desired reduction in CH_4_ emissions [11]. Additionally, calcium nitrate may improve nitrogen utilization by providing a readily available nitrogen source for microbial protein synthesis, thereby reducing nitrogen excretion and enhancing overall feed efficiency [6]. However, the effects of calcium nitrate on CH_4_ emission, nitrogen metabolism, and rumen fermentation dynamics in specific ruminant species, such as Liuyang black goats, remain understudied. As an important local breed in China, Liuyang black goats are known for their high meat quality and adaptability to local environments. Understanding the impact of calcium nitrate on these goats is crucial for developing sustainable feeding strategies that can reduce environmental impacts while maintaining or enhancing animal productivity.

The objective of this research was to investigate how dietary supplementation with calcium nitrate influences CH_4_ emissions, nitrogen excretion patterns, ruminal fermentation parameters, and the microbial community composition in Liuyang black goats. We hypothesized that adding calcium nitrate will not adversely affect the production health of goats, can reduce methane production, and optimize rumen fermentation. The findings will contribute to optimizing dietary strategies for environmentally sustainable goat production while maintaining animal health.

## 2. Materials and Methods

### 2.1. Animal, Diets and Treatments

We selected 12 male Liuyang Black goats sourced from the Subtropical Agricultural Research Institute of the Chinese Academy of Sciences in Changsha. The goats were randomly assigned to two groups: a control group (CON) and a treatment group (CAL) that was fed a diet containing 3% calcium nitrate (dry weight basis, grams per kilogram). Each group consisted of 6 goats with an average weight of 28 ± 0.2 kg; each goat served as an independent replicate. The experiment consisted of two 20-day periods, totaling 40 days. Feeding conditions remained consistent throughout the entire experiment. During the first 20 days, samples were collected from 3 goats in each group; during the second 20 days, samples were collected from the remaining 3 goats in each group. The calcium nitrate used in the treatment group was a commercial compound obtained from Rhus Chinensis Mill, with a purity of 99.9%, supplied by the National Pharmaceutical Chemical Reagent Company in Shanghai. Each phase included a 10-day pre-experimental period. On the 15th day of each phase, respiratory metabolism tests were performed using a single-chamber system, measuring the dynamic emissions of CH_4_ and CO_2_ over two consecutive days. Each goat was housed in a spacious, well-ventilated pen measuring 2.5 m in length and 1.5 m in width. They received feed two times a day, specifically at 8:00 AM and 5:00 PM, and they always had free access to clean drinking water.

The formulation of the experimental diet and the calculation of its nutritional components were based on the Agricultural Industry Standard of the People’s Republic of China (NY/T816-2021) [12], which outlines the nutritional requirements for male goat weighing approximately 30 ± 0.2 kg. The experimental diet consisted of fully mixed pellet feed with a diameter of approximately 3.5 mm and a length of 1 to 2 cm, provided by Gansu Runmu Biological Engineering Co., Ltd., Jinchang, China. Detailed information related to the composition and chemical analysis of the experimental diet can be found in Table 1.

### 2.2. Sample Collection

The experimental design comprised an initial 10-day adaptation period, succeeded by a 5-day sampling phase (days 11–15) in each phase. Sampling methods were adapted from Wang et al. [13]. During the sampling period, daily measurements had been conducted to gather essential data: (1) Daily records had been maintained for the quantity of feed provided and the residual amount to calculate dry matter intake (DMI). (2) Fecal samples were collected using plastic buckets placed behind the test sheep. (3) Urine output had been captured via a collection system featuring a PVC tube fitted onto the penis, draining into a storage bucket. All collected samples had undergone standardized processing procedures. Fecal samples had been homogenized, with 10% (*w*/*w*) of the aliquot reserved for further analysis. Urine output had been quantified by volume, and a 10% (*v*/*v*) subsample had been taken for additional examination.

Throughout the formal trial period, body weight (BW) measurements had been taken every morning prior to feeding. The goats’ average daily weight gain (ADG) was calculated by evaluating the differences between their starting and ending BW.

On the thirteenth day of each experimental phase, rumen fluid samples were collected using a rumen tube and vacuum pump prior to feeding, and again three hours after feeding. To avoid contamination from saliva, approximately 20 mL of the initial sample was discarded. The leftover fluid was subsequently filtered through four layers of gauze and examined right away for pH levels with the help of a portable pH meter (pH-HJ90, Beijing Aerospace Computer Co, Beijing, China). In order to facilitate additional analysis of ammonia nitrogen in conjunction with volatile fatty acids (VFAs), two samples were preserved at a temperature of −20 °C. Additionally, another two aliquots had been preserved at −80 °C for 16S rRNA pyrosequencing.

On the 15th day of each experimental phase following the goats’ adaptation to the respiration chamber, methane CH_4_ and carbon dioxide CO_2_ production was measured using a mobile open-circuit respiratory calorimeter system. The specific methodology followed the experimental protocol established by Wang et al. [14]. The measurement process had been designed to span two days and was conducted within an open respiration chamber, which maintains an average airflow rate of 40 m^3^/h as regulated by a gas flow meter (C100L-CRWE-DD, SIYA, Shanghai, China). During this period, a sophisticated greenhouse gas analyzer (MIU-374-8, Los Gatos Research, San Jose, CA, USA) had assessed gas concentrations at both the chamber outlet and in the surrounding environment at hourly intervals. Each measurement consisted of a 9 min detection phase followed by a 9 min control phase. Additionally, a gas flow meter (C100L-CRWE-DD, SIYA, Shanghai, China) had continuously monitored the gas production rate of the Liuyang black goats throughout the entire day. The data collected had been transmitted to a computer system for processing and calibration with pure CH_4_ and CO_2_ at a flow rate of 100 mL/min.

### 2.3. Chemical Analysis

Fecal samples were subjected to drying in an oven set at 60 °C for a duration of 48 h, after which they were ground with a vertical Wiley mill produced by Arthur H. Thomas Company located in Philadelphia, PA, USA, until they could pass through a sieve of 0.45 mm. The assessment of the chemical composition involved quantifying the DM content (utilizing method 930.15; AOAC, 2012), assessing CP levels (applying method 984.13; AOAC, 2012), and measuring the concentrations of calcium and phosphorus (methods 942.05 and 965.17; AOAC, 2012) [15]. The Ankom A200i fiber analyzer, developed by ANKOM Technology Co. (New York, NY, USA), was employed to evaluate acid detergent fiber (ADF) and neutral detergent fiber (NDF), following the protocols set forth by Van Soest et al. [16]. The calculation of digestible energy (DE) was conducted in line with the methods suggested by the National Research Council (NRC) and associated tables for goats [17].

To investigate the VFAs found in ruminal fluid, a high-performance gas chromatograph (HPGC; model GC-2014; produced by Shimadzu Corporation, Kyoto, Japan) was employed. This apparatus features a flame ionization detector (FID) that operates using hydrogen and includes a capillary column supplied by Agilent Technologies, Inc. (Wilmington, DE, USA). The column has a length of 30 m, an inner diameter of 0.32 mm, and a film thickness of 0.50 µm. The temperatures for both the column and FID were set at 120 °C and 200 °C, respectively, with high-purity nitrogen gas (99.99%) serving as the carrier gas at a flow rate of 1.2 mL/min. Hydrogen and air were delivered at rates of 30 mL/min and 300 mL/min, respectively. For the preparation of samples, 1 mL of ruminal fluid was combined with 0.25 mL of metaphosphoric acid (25%, *w*/*v*) and then centrifuged at 4 °C and 3000× *g* for 15 min to extract the supernatant for analysis. The ammonia nitrogen (NH_3_-N) concentration in the ruminal fluid was quantified using a UV/Visible spectrophotometer (UV-1801), manufactured by Beijing Beifen-Ruili Analytical Instrument Co, Ltd. (Beijing, China), in accordance with the procedure outlined by Broderick and Kang [18], utilizing the phenol-chloramine colorimetric method.

### 2.4. Sequencing of the 16S rRNA Gene and Bioinformatics Analysis

Samples of rumen content were obtained from six black goats in each experimental group. The experimental method referenced that of Yang et al. [19]. Genomic DNA was fully extracted with the QIAamp DNA Stool Mini Kit provided by QIAGEN (Hilden, Germany). The amplification of hypervariable regions V3 and V4 of the bacterial 16S rRNA gene was executed using barcode-labeled primers 515F (5′-GTGCCAGCMGCCGCGGTAA-3′) and 806R (5′-GGACTACHVGGGTWTCTTAAT-3′) [20,21]. The PCR reaction consisted of 15 μL of Phusion^®^ High-Fidelity PCR Master Mix (New England Biolabs, Ipswich, MA, USA), along with 0.2 μM of each primer and 10 ng of template DNA. The thermal cycling protocol included an initial denaturation step at 98 °C for one minute, followed by 30 cycles of annealing and extension, concluding with a final extension at 72 °C for a duration of 5 min [22]. The size of the amplified products was assessed using 2% agarose gel electrophoresis and then purified with the Qiagen gel extraction kit (QIAGEN GmbH, Hilden, Germany). Finally, paired-end sequencing of 250 bp was conducted on the Illumina NovaSeq platform (Illumina, San Diego, CA, USA). The assembly of raw reads was carried out employing FLASH software (Version 1.2.7, http://ccb.jhu.edu/software/FLASH/ (accessed on 3 April 2025)) [23]. Subsequently, QIIME (Version 1.9.1, http://qiime.org/scripts/split_libraries_fastq.html (accessed on 3 April 2025)) [24] was employed for quality control to eliminate chimeric sequences, resulting in effective tags. The sequences were subsequently grouped into operational taxonomic units (OTUs) based on a similarity threshold of 97% [25]. The UPARSE tool (Version 7.0.1001, http://www.drive5.com/uparse/ (accessed on 3 April 2025)) was utilized, with species classification based on the UCHIME algorithm [26]. Furthermore, the Chao1 and Shannon diversity indices were computed [27], while principal coordinates analysis (PCoA) and visualization were performed utilizing R software (version 3.5.2, R Foundation for Statistical Computing, Vienna, Austria) [28]. Lastly, taxonomic classification was conducted using Mothur software (version 1.41.1), referencing the Silva.nr.132 database [29]. We performed linear discriminant analysis effect size (LEfSe) analysis with the Micro Biome Process package (version 1.1.463, RStudio, Inc., Boston, MA, USA) in RStudio (version 3.5.2, R Foundation for Statistical Computing, Vienna, Austria) to evaluate all classification levels ranging from phylum to species, establishing a threshold for LDA scores (≥3.5) to ascertain the significance of the identified biomarkers. Additionally, Spearman rank correlation analysis was applied to examine the relationship between rumen fermentation characteristics and the relative abundances of the top 10 dominant bacterial genera and phyla.

### 2.5. Calculations and Statistical Analysis

The CH_4_ emission, CO_2_ emission, growth performance and apparent nutrient digestibility were statistically analyzed with the aid of SPSS software (IBM Corp. Released 2019, IBM SPSS Statistics for Windows, Version 26.0, Armonk, NY, USA: IBM Corp.). The linear model applied was organized in the following manner:

*Y_i_* = *μ* + *T_i_* + *β_t_⋅t_j_* + *ε_i_*

In this context, *Y*i represents the mean treatment observed during the 5-day collection period for the ith dietary group. The overall mean is indicated by *μ*. The fixed effects of the two treatments (CON and CAL, corresponding to *i* = 1 and 2) are denoted by *T*_i_. The term *β*_t_ denotes the effect associated with the time stage, illustrating how time influences the outcome variable. The time covariate, *t*_j_, is defined such that *t*_j_ = 1 corresponds to the initial time stage, while *t*_j_ = 2 refers to the subsequent time stage. Lastly, *ε*_i_ represents the random residual error.

SPSS software (IBM Corporation 2019 edition, IBM SPSS Statistics for Windows, Version 26.0, Armonk, NY, USA: IBM Corporation) was used to examine the normality assumption, homogeneity of variance, and outlier handling for data on average daily gain (ADG), apparent nutrient digestibility, rumen fermentation indicators, and relative abundance of rumen bacteria. Independent samples *t*-tests were performed. The models used are as follows:

$$t=\frac{\bar{x_{1}}-\bar{x_{2}}}{S_{\bar{x_{1}}-\bar{x_{2}}}}$$

In this context, $\bar{x_{1}}$ and $\bar{x_{2}}$ denote the average values of various treatment groups, whereas $S_{\bar{x_{1}}-\bar{x_{2}}}$ represents the standard error associated with the mean difference. A significance threshold of *p* < 0.05 was determined. Additionally, Spearman’s correlation coefficients were utilized to assess the associations between microbial species and rumen VFAs, with the same statistical significance threshold of *p* < 0.05 applied.

## 3. Results

### 3.1. Effects of Dietary Calcium Nitrate Supplementation on Methane and Carbon Dioxide Emissions

**Figure 1:**
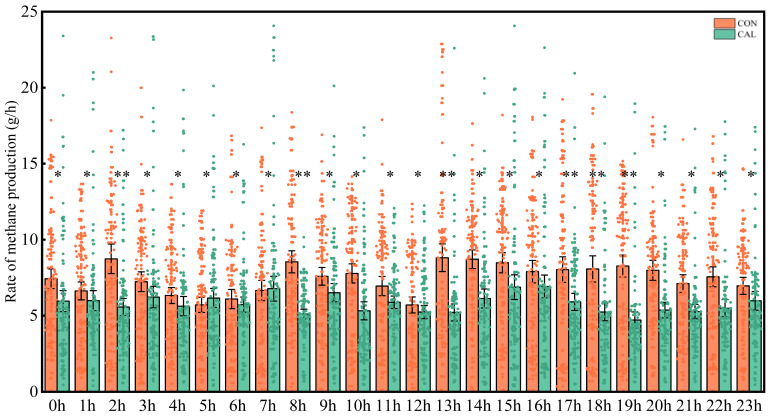
Diurnal pattern in CH_4_ emission in respiration chambers (g/h). At the same time, * above the histogram indicated significant difference (*p*< 0.05), and ** indicated extremely significant difference (*p* ≤ 0.01). Abbreviation: CON = control group; CAL = calcium nitrate group.

**Figure 2:**
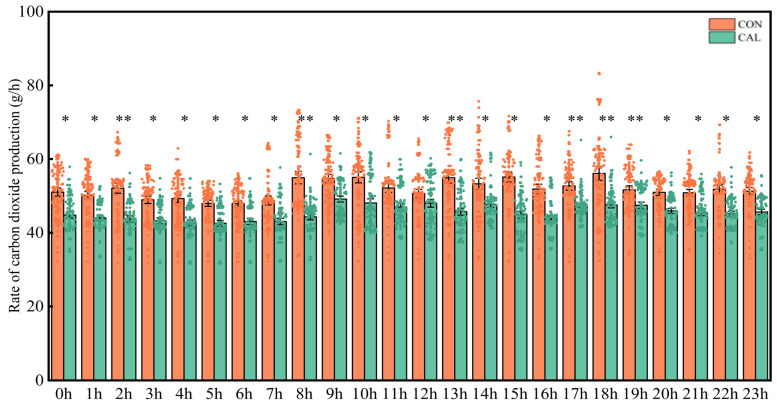
Diurnal pattern in CO_2_ emission in respiration chambers (g/h). At the same time, * above the histogram indicated significant difference (*p* < 0.05), and ** indicated extremely significant difference (*p* ≤ 0.01). Abbreviation: CON = control group; CAL = calcium nitrate group.

The emissions of CH_4_ and CO_2_ were tracked and visually displayed (Figure 1 and Figure 2). The findings indicated that the CAL group had consistently lower emissions of both CH_4_ and CO_2_ compared to the CON group across all time points measured (*p* < 0.05). Specifically, inclusion of calcium nitrate in the diet led to a significant decrease in the emission rates of CH_4_ and CO_2_ in goats (*p* < 0.05). Furthermore, the temporal patterns of CH_4_ and CO_2_ production in goats exhibited a distinct diurnal variation, with emissions peaking within 2 to 3 h after feeding and subsequently decreasing until the next feeding event.

The diet of goats that included calcium nitrate led to a noteworthy reduction in daily CH_4_ emissions, as shown in Table 2. Specifically, the production of CH_4_ per unit of dry matter intake (DMI) was significantly lower in the CAL group when compared to the CON group (CH_4_/DMI, *p* < 0.05). Conversely, no significant differences were observed in methane emissions per unit of average daily gain between the two groups (CH_4_/ADG, *p* > 0.05). Similarly, methane emissions per unit of neutral detergent fiber intake did not differ significantly between the groups (CH_4_/NDFI, *p* > 0.05). Additionally, no significant differences were found in methane emissions per unit of acidic detergent fiber intake (CH_4_/ADFI, *p* > 0.05). Moreover, incorporating calcium nitrate into the diet resulted in significant decreases in both CH_4_ energy loss and emissions per metabolic BW relative to the CON group (CH_4_-E, CH_4_/BW^0.75^, *p* < 0.05). Similarly, the CAL group showed substantially lower daily CO_2_ emissions compared to the CON group (*p* < 0.05). In addition, the CAL group exhibited significant reductions across all individual indicators of CO_2_ emissions (*p* < 0.05).

### 3.2. Effects of Dietary Calcium Nitrate Supplementation on Apparent Nutrient Digestibility and Growth Performance

As shown in Table 3, the addition of calcium nitrate to the diets of goats did not significantly impact their growth performance or nutrient digestibility (*p* > 0.05). Calcium nitrate supplementation, in particular, did not have a significant impact on ADG, feed conversion ratio (F/G), or DMI (*p* > 0.05). However, the apparent nutrient digestibility was reduced with calcium nitrate supplementation, even though the differences between the supplemented group and the control group were not statistically significant (*p* > 0.05).

### 3.3. Effects of Dietary Calcium Nitrate Supplementation on Nitrogen Metabolism

Table 4 presents the effects of calcium nitrate supplementation on nitrogen metabolism in goats. The data indicated that dietary calcium nitrate did not significantly influence nitrogen intake, faecal nitrogen, urine nitrogen, digestive nitrogen, nitrogen deposition, nitrogen digestibility, net protein utilization, or the biological value of proteins in goats (*p* > 0.05).

### 3.4. Effects of Dietary Calcium Nitrate Supplementation on Rumen Fermentation Parameters

Table 5 presents the effects of calcium nitrate supplementation on ruminal pH, NH_3_-N, and VFAs in goats. Before feeding, no significant differences were observed between the two groups in terms of pH, NH_3_-N, total volatile fatty acids (TVFAs), the proportion of individual VFAs, and the acetate to propionate ratio (A:P) (*p* > 0.05). However, a significant difference was observed in valerate proportions (*p* < 0.05), suggesting a specific effect of calcium nitrate on this VFAs.

Three hours after feeding, significant differences in rumen fermentation patterns were observed in goats. The CAL group had a higher pH compared to the CON group (*p* < 0.05). Additionally, NH_3_-N production increased, but no significant difference was observed (*p* > 0.05). The CAL group also exhibited a lower acetate proportion and higher propionate proportion, along with increased concentrations of valerate, isobutyrate, and isovalerate (*p* < 0.05). These findings suggest that calcium nitrate supplementation modulates ruminal fermentation patterns, favoring propionate production over acetate (*p* < 0.05).

### 3.5. Effects of Dietary Calcium Nitrate Supplementation on Diversity of Rumen Bacterial Community

**Figure 3:**
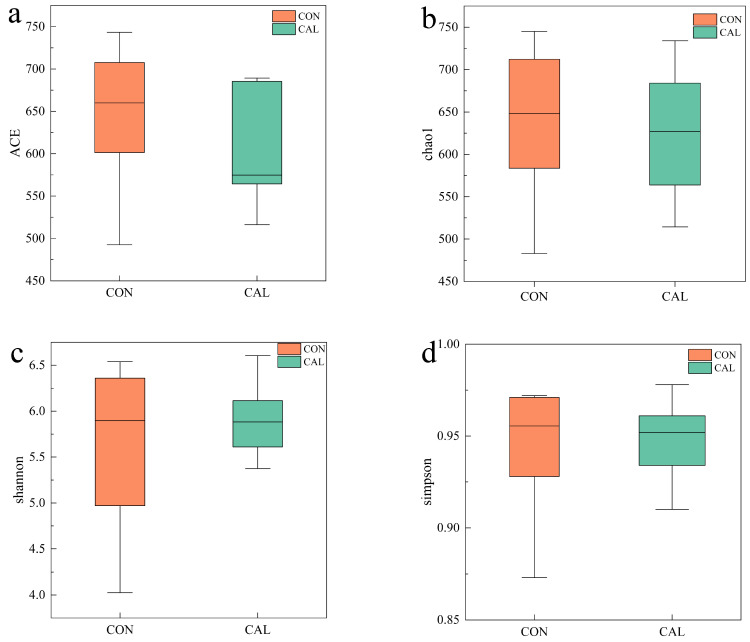
The microbial community’s alpha diversity in the rumen is influenced by the species observed. The indexes used to measure alpha diversity comprise (**a**) the ACE index, (**b**) the chao1 index, (**c**) the Shannon index, and (**d**) the Simpson index. Abbreviations used include: CON = control group; CAL = calcium nitrate group.

**Figure 4:**
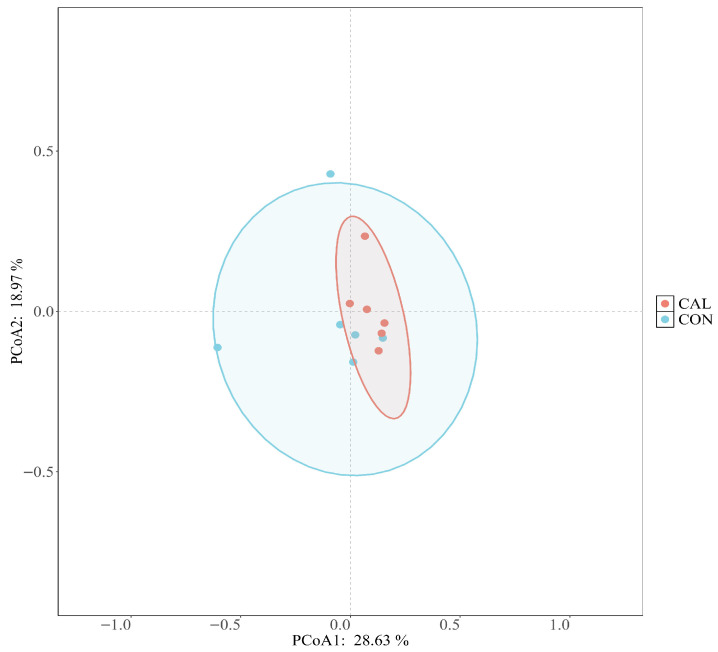
A principal coordinate analysis (PCoA) was performed to illustrate the makeup of the rumen bacterial community subjected to different treatments, Abbreviations: CON = control group; CAL = calcium nitrate group.

The incorporation of calcium nitrate did not significantly influence the alpha diversity metrics, such as ACE, Chao1, Shannon, and Simpson, related to the bacterial microflora found in the rumen fluid of Liuyang black goats (*p* > 0.05, Figure 3). Meanwhile, the beta diversity assessment utilizing weighted UniFrac distances showed a more centralized spatial arrangement of samples within the CAL group, suggesting enhanced internal consistency. In contrast, the CON group displayed a more dispersed pattern. This suggests that the CAL group exhibited a significantly higher degree of sample aggregation compared to the CON group (Figure 4).

**Figure 5:**
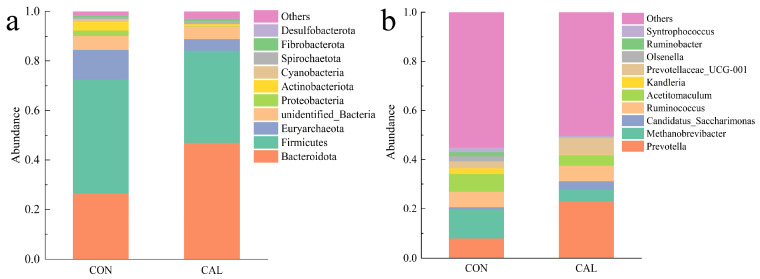
The structure of the rumen microbiome at both the phylum and genus levels: (**a**) the composition of rumen microbiome at phylum level, (**b**) the composition of major rumen genus level; Abbreviations: CON = control group; CAL = calcium nitrate group.

Among bacteria, the study’s findings indicated that the microbial community composition and relative abundance varied betwen the two treatment groups, with *Bacteroidota* and *Firmicutes* being the dominant phyla. Notably, *Bacteroidota* was most abundant in the CAL group and *Firmicutes* in the CON group (*p* < 0.05, Figure 5a and Table 6). At the genus level, *Prevotella* and *Methanobrevibacter* were particularly prominent, with *Prevotella* showing a higher relative abundance in the CAL group and *Methanobrevibacter* in the CON group (*p* < 0.05, Figure 5b and Table 7), and some genera such as *Prevotellaceae_UCG-001* appeared to be more abundant in the CAL group compared to the CON group (*p* < 0.05).

**Figure 6:**
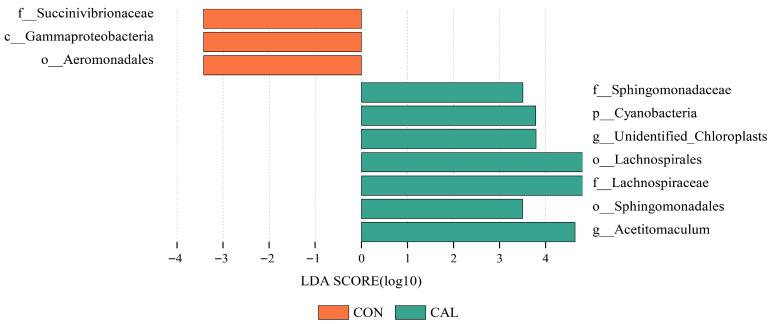
The comparison of the microbial community composition in goat’s rumen using the LEfSe analysis (LDA = 3.5); Abbreviations: CON = control group; CAL = calcium nitrate group; LDA = linear discriminant analysis.

The analysis using Linear discriminant analysis effect size (LEfSe) demonstrated clear differences in the characteristics of the microbial communities between the various treatment groups. In the rumen, the CAL group was significantly enriched with a diverse array of taxa, including *Sphingomonadaceae*, Unidentified_chloroplasts, *Lachnospirales*, *Lachnospiraceae*, and *Sphingomonadales*. In contrast, the CON group showed enrichment with three primary taxa, including *Succinivibrionaceae*, *Gammaproteobacteria*, and *Aeromonadales* (Figure 6).

**Figure 7:**
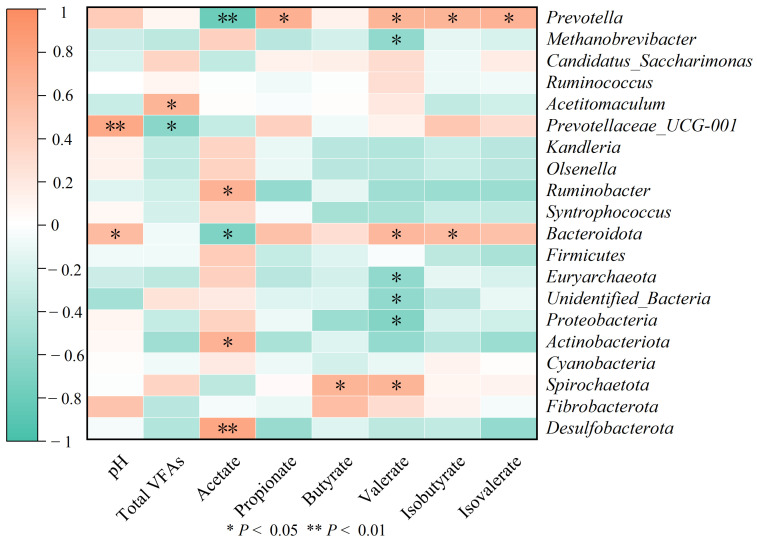
Analysis of the Spearman correlation between microbiota at the genus and phylum levels and parameters of rumen fermentation. * *p* < 0.05. ** *p* < 0.01.

Spearman correlation analysis revealed meaningful relationships between parameters of rumen fermentation and the relative abundance of various microbial taxa (Figure 7). The pH level in the rumen was positively correlated with the abundance of *Bacteroidota* (*p* < 0.05). The concentration of acetate was positively associated with *Ruminobacter* (*p* < 0.05), *Actinobacteriota* (*p* < 0.05), and *Desulfobacterota* (*p* < 0.01), whereas it showed negative correlations with *Bacteroidota* (*p* < 0.05) and *Prevotella* (*p* < 0.01). Propionate concentration was positively associated with *Prevotella* abundance (*p* < 0.05). Butyrate and valerate concentrations both correlated positively with *Spirochaetota* (*p* < 0.05). The TVFA showed a significant positive relationship with *Acetitomaculum* abundance (*p* < 0.05).

## 4. Discussion

CH_4_ is responsible for about 16% of worldwide human-made greenhouse gas emissions and has a significantly greater warming potential compared to CO_2_ [30]. The production of ruminal CH_4_ is a multifaceted trait affected by various factors, such as DMI, the composition of feed, the microbiota present in the rumen, the ratio of fermentation byproducts, genetic traits of the host, and environmental influences [31]. Upon entering the rumen of ruminants, cellulose undergoes fermentation, yielding VFAs and reduced H_2_. Within the rumen ecosystem, methanogenic archaea efficiently utilize the reduced H_2_ produced by other microorganisms through an interspecies H_2_ transfer mechanism [32]. Research indicates that CH_4_ production exhibits a gradual decline trend as nitrate supplementation in ruminant diets increases [33]. Research has shown that incorporating NO_3_^−^ into ruminant diets can effectively reduce CH_4_ emissions in vivo, with observed reduction potentials ranging from 6.8% to 12.5% for each 1% of NO_3_^−^ added on a dry DM basis per day [34]. Asanuma et al. [35] reported a sharp decrease in the number of methanogens in the rumen of goats following the addition of nitrate, suggesting that nitrate may be toxic to methanogens, thereby reducing CH_4_ emissions by inhibiting their activity. However, findings on the effects of nitrate on CH_4_ emissions are not consistent across studies. Meller et al. [36] and Rebelo et al. [37] observed reduced CH_4_ emissions with dietary nitrate supplementation but noted no significant differences in CH_4_ production. This difference is mainly linked to reduced DMI resulting from the nitrate effect, as there is a positive correlation between feed intake and intestinal CH_4_ emissions. In this study, the reduction in methane emissions was accompanied by no significant differences in DMI among treatment groups. Specifically, the dietary addition of 3% calcium nitrate significantly reduced CH_4_ production, which is in agreement with the findings of the aforementioned researchers. There was no significant difference in methane emissions per unit of acid-detergent fiber intake, which may be attributed to the influence of individual variation. Furthermore, the lack of data on methemoglobin in this study represents a limitation, and there may be differences in the safety thresholds across different species, highlighting the need for further studies on the effects of nitrate in the rumen.

In the present study, DMI and ADG were slightly higher in the CAL group compared to the CON group, although no significant differences were observed between the two groups. Meller et al. [36] and Rebelo et al. [37] noted that nitrate addition decreased DMI by approximately 8.0% at similar levels of inclusion, which was initially attributed to nitrate toxicity, characterized by elevated blood methemoglobin levels exceeding 20% of total hemoglobin. When animals were gradually acclimated to nitrate or protective nitrates were used, the decrease in DMI was primarily due to the bitter taste of nitrates, rather than toxicity. This suggests that gradual acclimatization is crucial for maintaining DMI levels without compromising animal performance or health. For instance, Halmemies et al. [9] fed growing goats (initial BW of 10 kg) diets supplemented with 5.0% calcium nitrate or 2.6% urea for 12 weeks and observed that growth rates were comparable when calcium nitrate was used as the main nitrogen source. Similarly, Van Zijderveld et al. [38] found no detrimental effects on DMI and ADG when crossbred Texel lambs (initial BW 43 kg) were fed 26 g/kg DM calcium nitrate for 4 weeks. Seyyedsalehi et al. [39] concluded that DMI reductions and signs of nitrate toxicity only occurred when diets contained more than 30 g/kg nitrate for sheep and more than 10 g/kg nitrate for beef cattle. Li et al. [10] also demonstrated that, compared to urea, nitrate (21 g/kg DM) did not significantly affect DMI but resulted in a 19% decrease in ADG, highlighting the need for further studies on the effects of nitrate in the rumen.

Enhancing the efficiency of microbial protein production in the rumen markedly reduces the urinary excretion of nitrogen-containing metabolites. The mechanism underlying this process is the efficient utilization properties of microbial proteins during digestion, which enable the nitrogen in their metabolites to be fully absorbed by the host, thereby minimizing urinary nitrogen losses [40]. In this investigation, significant differences were not found between the CAL and CON groups regarding nitrogen metabolism. These results align with those presented by Li et al. [10], who indicated that the inclusion of nitrate did not affect the digestibility of DM or nitrogen (N) when compared to that of control diets. The findings from the current study further suggest that nitrate may act as a nitrogen source for microbial development in the rumen. Thus, it is logical to propose that nitrate is efficiently utilized as a nitrogen source for microbial growth within the rumen. Additionally, Almeida et al. [41] noted an increase in rumen microbial nitrogen outflow from 9.3 to 11.8 g N daily as the dietary nitrate concentration was elevated from 0% to 4%. Similarly, some researchers have found that sheep fed nitrate-supplemented diets consumed more N compared to those fed urea-supplemented diets. However, this increased intake did not affect the apparent digestibility of N in sheep [42]. When considering energy efficiency, energy losses through CH_4_ were reduced by 3.5% in sheep consuming nitrate-supplemented diets. Although the proportion of energy lost as CH_4_ was lower for nitrate-added diets, no significant differences in metabolic energy intake were observed between treatments.

The rumen is a central component of the digestive system in ruminants, playing a vital role in the degradation of fibrous feeds and serving as the primary site for the absorption of key nutrients, such as proteins and trace elements [43]. Maintaining a healthy and balanced rumen environment is crucial for optimizing digestive efficiency and nutrient absorption in ruminants. The pH of rumen fluid is a key indicator of the fermentation environment within the rumen, with changes in pH directly reflecting the conditions of fermentation. The normal pH range for rumen fluid is typically 6.0 to 7.0 [44]. Following feeding, ruminal pH typically decreases and then gradually increases due to the absorption of VFAs, rumination, and salivation [45]. The present study also observed this pattern of pH fluctuation. In contrast to our results, Mahmoudi et al. [46] did not find a significant difference in rumen fluid pH when dietary nitrate was introduced for fattening rams. Nevertheless, other research has yielded contrasting outcomes. Ungerfeld et al. [47] detected a notable increase in rumen fluid pH with the supplementation of nitrate in an in vitro batch culture. Likewise, Hassan et al. [48] reported that incorporating nitrate into diets at concentrations of 6.84 g/kg DM and 6.80 g/kg DM led to higher pH levels. Additionally, an in vitro experiment by Zhou et al. [49] revealed that significant pH increases occurred when nitrate levels exceeded 24 μmol/L. These findings align with those of the current experiment.

NH_3_-N is released by rumen microorganisms during the decomposition of nitrogenous substances in feed. It serves not only as a key nitrogen source during rumen microbial fermentation but also as a fundamental indicator for assessing the efficiency of converting feed nitrogen to microbial nitrogen [50]. In the current study, the concentrations of NH_3_-N rose at 3 h after feeding when compared to pre-feeding levels, though no significant differences were found among the groups. The CAL group’s NH_3_-N concentration was greater than that of the CON group, mainly because calcium nitrate was included in the diet, resulting in nitrite production and subsequently higher rumen NH_3_-N levels [51]. Almeida et al. [41] noted that incorporating nitrate into the diet enhanced NH_3_-N production in the rumen, which aligns with the findings of this study. However, previous in vitro studies have shown that nitrate supplementation had no effect on NH_3_-N concentration [52,53]. Conversely, other reports indicate that nitrate supplementation may reduce NH_3_-N concentrations [54]. These conflicting results suggest that nitrate metabolism in the rumen is not exclusively converted to NH_3_, and its impact on NH_3_-N levels may vary depending on the experimental conditions and dietary formulations.

Ruminants derive 60% to 80% of their energy from VFAs produced through rumen digestion of nutrients [55]. Under standard conditions, the minimum hydrogen requirement for acetate synthesis by acetogenic bacteria is approximately 10 to 100 times higher than that for methanogenesis by methanogenic archaea [56]. If ruminal VFA production is shifted towards propionate (lower acetate-to-propionate ratio, A:P), the net balance of H_2_ in the rumen is reduced, thereby decreasing CH_4_ production [57]. Research indicates that the supplementation of nitrate enhances the growth of specific bacteria that degrade fiber in the rumen, as well as boosts the production of valeric acid within this environment [58]. The impact of nitrate supplementation on the composition of VFAs has shown variability among different studies. For instance, Feng et al. [57] observed a linear increase in the proportion of propionic acid with the administration of three calcium nitrate doses at 5.3, 13.6, and 21.1 g/kg DM, a finding that aligns with the conclusions of the present study. This variation may be attributed to differences in sampling time and the dose-dependent effect of nitrate supplementation on CH_4_ mitigation [57]. Numerous investigations highlighted a notable impact of nitrate supplementation on the profiles of VFAs, with sampling often conducted shortly after feeding—especially in grazing cows given dietary nitrate at concentrations of 22.5, 21.5, and 18 g/kg DM [59]. Conversely, Li et al. [10] reported no influence of nitrate supplementation on rumen VFAs when samples were taken from goats over six hours post-feeding. In our current study, we noted alterations in acetate proportions due to treatment and a marked reduction in the A:P ratio, which somewhat diverges from certain earlier findings [60]. This reduction was linked to an increase in H_2_ release and a boost in the abundance and functioning of microorganisms that produce acetate [51]. Moreover, a noteworthy decrease in the A:P ratio was noted in the CAL group in contrast to the CON group, which could be linked to changes in the fermentation process resulting from variations in the relative abundance of rumen microorganisms, including *Fibrobacterium* and *Ruminococcus* species.

In the current study, it was observed that the relative abundance of the methanogenic genus *Methanobrevibacter* diminished at the genus level. Bharanidharan et al. [61] similarly noted that the addition of NO_3_^−^ resulted in a 54% decrease in the population of methanogenic bacteria in ruminants. Furthermore, Li et al. [10] indicated that nitrate could significantly lower both *Methanobrevibacter* levels and CH_4_ emissions in sheep. Other research has also indicated that nitrate supplementation helps reduce the numbers of methanogenic bacteria, consequently leading to a decrease in CH_4_ production during fermentation. This has important implications for controlling greenhouse gas emissions from ruminants and reducing the energy losses associated with CH_4_ emissions from animal feeds, which can range from 2% to 12% [62]. Furthermore, these studies suggest that nitrates can exert long-term effects on rumen methanogenic bacteria [63,64]. In the rumen, CH_4_ is primarily produced by methanogenic archaea, which use H_2_ and CO_2_ as substrates for CH_4_ synthesis [65]. When nitrates enter the rumen, nitrate-reducing bacteria utilize H_2_ as an electron donor and nitrate ions as an electron acceptor. Through the action of nitrate reductase, nitrates are reduced to nitrites [66]. During this reduction process, nitrates compete with methanogens for electrons from H_2_ donors, thereby inhibiting CH_4_ production, reducing greenhouse gas emissions, and minimizing feed energy waste [67]. Patra and Yu [68] observed that nitrate has a high electron affinity, which allows it to outcompete methanogens for electrons, thereby further inhibiting CH_4_ production.

## 5. Conclusions

The research illustrated that the supplementation of dietary calcium nitrate in Liuyang black goats led to a significant decrease in CH_4_ and CO_2_ emissions, thus emphasizing its potential as a method for reducing greenhouse gas emissions from ruminant animals. Additionally, the results indicated that calcium nitrate influenced ruminal fermentation dynamics and modified the makeup of the rumen microbiota. These findings highlight the possible advantages of using calcium nitrate as a feed additive to enhance the sustainability of goat production systems. Future investigations should aim to refine the dosage of calcium nitrate and examine its long-term implications for animal health, productivity, and overall environmental effects.

## Figures and Tables

**Table 1:** Composition and nutrition level of the experiment diets (DM basis).

Items	Treatments ^1^	
	CON	CAL
Ingredients, %		
Corn straw	17.76	17.76
Soy husk	10.34	10.34
Sunflower hull	2.00	2.00
Corn	48.60	49.65
Soybean meal	11.35	7.55
Sesame cake	3.00	3.00
Molasses	5.00	5.00
Salt	0.70	0.70
Expanded urea	0.80	0.00
Calcium carbonate	0.25	0
Premix ^2^	1.00	1.00
Calcium nitrate	0.00	3.00
Total	100.00	100.00
Nutrient level ^3^		
DE (MJ/kg)	12.31	11.92
CP (%)	14.34	14.70
EE (%)	2.48	2.95
NDF (%)	39.22	38.09
ADF (%)	17.20	16.89
Ca (%)	0.31	0.81
P (%)	0.21	0.22

^1^ Treatments; control group (CON); calcium nitrate group (CAL). ^2^ Premix ingredients: Fe = 25 mg, Mn = 40 mg, Zn = 40 mg, Cu =8 mg, I = 0.3 mg, Se = 0.3 mg, Co = 0.1 mg, Vit A = 940 IU, Vit D = 111 IU, Vit E = 20 IU. ^3^ Digestible energy (DE), the digestive energy in the nutritional level was calculated, and the rest were measured; crude protein (CP), ether extract (EE), neutral detergent fiber (NDF), acid detergent fiber (ADF), calcium (Ca), phosphorus (P).

**Table 2:** Effects of calcium nitrate on methane emission in goats.

Items ^1^	Treatment ^2^		SEM ^3^	*p*-Value
	CON	CAL		
**CH** **_4_**				
CH_4_ (g/d)	13.738	6.272	1.73	0.041
CH_4_ (g/kg DMI)	17.704	8.029	1.19	0.036
CH_4_ (g/kg ADG)	75.251	28.936	1.31	0.055
CH_4_ (g/kg NDFI)	33.997	19.077	1.88	0.072
CH4 (g/kg ADFI)	49.461	39.828	4.19	0.283
CH_4_-E (MJ/d)	0.765	0.349	0.10	0.041
CH_4_/BW^0.75^	1.372	0.644	0.17	0.042
**CO** **_2_**				
CO_2_ (g/d)	546.606	275.253	50.90	0.007
CO_2_ (g/kg DMI)	713.616	352.072	62.87	0.001
CO_2_ (g/kg ADG)	3133.288	1267.421	407.05	0.013
CO_2_ (g/kg NDFI)	1308.544	836.508	91.80	0.003
CO_2_ (g/kg ADFI)	2934.239	1886.476	204.63	0.003
CO_2_-E (MJ/d)	4.888	2.462	0.46	0.007
CO_2_/BW^0.75^	54.248	28.177	4.66	0.003

^1^ Items; CH_4_, daily methane emission; CH_4_/DMI, methane emission from dry matter intake; CH_4_/ADG, methane emission from average daily gain; CH_4_/NDFI, methane emission per neutral detergent fiber intake; CH_4_/ADFI, methane emission per acid detergent fiber intake; CH_4_-E, methane energy; BW^0.75^, metabolic body weight; CO_2_, daily carbon dioxide emission; CO_2_/DMI, carbon dioxide emission from dry matter intake; CO_2_/ADG, carbon dioxide emission from average daily gain; CO_2_/NDFI, carbon dioxide emission per neutral detergent fiber intake; CO_2_/ADFI, carbon dioxide emission per acid detergent fiber intake; CO_2_-E, carbon dioxide energy. ^2^ Treatments; control group (CON); calcium nitrate group (CAL). ^3^ SEM, standard error of means for treatments.

**Table 3:** Effects of calcium nitrate addition on apparent digestibility of nutrients in goats.

Items	Treatment ^2^		SEM ^3^	*p*-Value
	CON	CAL		
Growth performance ^1^				
DMI, g/d	764.750	784.108	29.62	0.761
ADG, g/d	193.770	217.725	49.46	0.423
F/G	4.674	3.998	0.30	0.297
Apparent nutrient digestibility, %				
DM	40.863	35.134	0.03	0.374
NDF	45.714	39.549	0.03	0.258
ADF	24.483	19.064	0.03	0.385
CP	67.502	62.487	0.02	0.188

^1^ Growth performance; DMI, dry matter intake; ADG, average daily gain; F/G, feed to gain ratio DM, dry matter; NDF, neutral detergent fiber; ADF, acid detergent fiber; CP, crude protein. ^2^ Treatments; control group (CON); calcium nitrate group (CAL). ^3^ SEM, standard error of means for treatments.

**Table 4:** Effects of calcium nitrate addition on nitrogen metabolism in goats.

Items	Treatment ^1^		SEM ^2^	*p*-Value
	CON	CAL		
Nitrogen intake, g/d	18.365	17.153	0.58	0.321
Faecal nitrogen, g/d	6.373	6.955	0.39	0.493
Urine nitrogen, g/d	6.954	6.255	0.55	0.553
Digestive nitrogen, g/d	11.993	10.200	0.56	0.113
Nitrogen deposition, g/d	5.039	3.945	0.31	0.074
Nitrogen digestibility, %	65.325	59.343	0.02	0.149
Net protein utilization, %	27.887	22.900	0.02	0.165
Biological value of protein, %	43.567	38.433	0.03	0.436

^1^ Treatments; control group (CON); calcium nitrate group (CAL). ^2^ SEM, standard error of means for treatments.

**Table 5:** Effect of calcium nitrate on ruminal pH, NH_3_-N, VFAs in goats.

Items	Treatment ^1^		SEM ^2^	*p*-Value
	CON	CAL		
Before feeding				
pH	6.563	6.683	0.15	0.489
NH_3_-N, mg/100 mL	10.809	12.044	0.68	0.378
TVFAs ^3^, mmol	58.360	67.318	4.45	0.325
VFA proportion, mol/100 mol				
Acetate	60.266	62.099	0.78	0.249
Propionate	19.647	17.814	0.95	0.350
Butyrate	15.686	15.742	0.44	0.950
Valerate	0.755	1.055	0.73	0.045
Isobutyrate	1.374	1.241	0.07	0.361
Isovalerate	2.273	2.049	0.11	0.316
Acetate/Propionate	3.354	3.567	0.17	0.551
3 h after feeding				
pH	5.838	6.290	0.15	0.022
NH_3_-N, mg/100 mL	13.461	15.434	0.72	0.184
TVFAs ^3^, mmol	111.159	101.901	4.60	0.325
VFA proportion, mol/100 mol				
Acetate	62.753	53.014	1.51	0.001
Propionate	21.718	32.354	1.52	0.001
Butyrate	13.120	13.112	0.40	0.917
Valerate	0.696	1.022	0.07	0.014
Isobutyrate	0.295	0.560	0.04	0.001
Isovalerate	0.441	0.835	0.06	0.001
Acetate/Propionate	3.033	1.747	0.20	0.001

^1^ Treatments; control group (CON); calcium nitrate group (CAL). ^2^ SEM, standard error of means for treatments. ^3^ TVFAs, Total volatile fatty acids.

**Table 6:** Relative abundance of rumen microorganisms (phylum level, %).

Items	Treatments ^1^		SEM ^2^	*p*-Value
	CON	CAL		
*Firmicutes*	46.107	37.168	0.03	0.180
*Bacteroidota*	26.483	47.036	0.04	0.007
*Euryarchaeota*	11.911	4.664	0.02	0.094
Unidentified_Bacteria	5.677	5.057	0.02	0.871
*Actinobacteriota*	3.410	0.849	0.01	0.173
*Proteobacteria*	2.123	0.141	0.01	0.366
*Spirochaetota*	0.841	1.227	0.01	0.436
*Fibrobacterota*	0.568	0.814	0.01	0.473
*Cyanobacteria*	1.199	0.062	0.01	0.377
*Desulfobacterota*	0.130	0.092	0.01	0.272

^1^ Treatments; control group (CON); calcium nitrate group (CAL). ^2^ SEM, standard error of means for treatments.

**Table 7:** Relative abundance of rumen microorganisms (genus level, %).

Items	Treatments ^1^		SEM ^2^	*p*-Value
	CON	CAL		
*Prevotella*	7.970	22.952	0.03	0.001
*Methanobrevibacter*	11.860	4.644	0.02	0.005
*Ruminococcus*	6.330	6.295	0.01	0.991
*Acetitomaculum*	7.146	4.276	0.01	0.254
*Prevotellaceae_UCG-001*	2.840	7.115	0.01	0.044
*Candidatus_Saccharimonas*	0.764	3.579	0.02	0.434
*Kandleria*	2.358	0.162	0.01	0.367
*Syntrophococcus*	1.899	0.445	0.01	0.335
*Olsenella*	2.117	0.150	0.01	0.327
*Ruminobacter*	1.633	0.018	0.01	0.363

^1^ Treatments; control group (CON); calcium nitrate group (CAL). ^2^ SEM, standard error of means for treatments.

## Data Availability

The data will be made available from the corresponding author upon reasonable request.

## Abbreviations

In this manuscript, the abbreviations listed below are utilized:

CAL	Calcium Nitrate Group
CON	Control Group
CH_4_	Methane
NH_3_-N	Ammonia Nitrogen
A:P	Acetate-to-propionate Ratio
VFAs	Volatile Fatty Acids
CO_2_	Carbon Dioxide
GHGs	Greenhouse Gases
ADG	Average Daily Weight Gain
BW	Body Weight
